# Transdermal Delivery of Salbutamol from Nanoemulsions: Influence of Ternary Composition and Surfactant Architecture

**DOI:** 10.3390/pharmaceutics18091127

**Published:** 2026-09-08

**Authors:** Özge Esen Yigit, Alf Lamprecht

**Affiliations:** 1 Department of Pharmaceutics, Institute of Pharmacy, University of Bonn, 53121 Bonn, Germany; s6oeesen@uni-bonn.de; 2 EFS, INSERM UMR1098 RIGHT, Université Marie et Louis Pasteur, F-25000 Besançon, France

**Keywords:** transdermal drug delivery, nanoemulsions, hydrophilic drug delivery, ternary composition, skin model selection

## Abstract

**Background/Objectives**: Transdermal delivery of hydrophilic drugs remains limited by poor partitioning into the lipid-rich stratum corneum (SC). This study systematically investigated how ternary nanoemulsion composition and surfactant architecture jointly influence the transdermal delivery of salbutamol and whether the resulting composition–performance relationships are preserved across different skin models. **Methods**: Salbutamol-loaded nanoemulsions were prepared by the phase inversion temperature (PIT) method across a predefined ternary design space using two non-ionic surfactant systems: polyoxyl castor oil and polyoxyl hydroxystearate. Physicochemical characterization, ternary compositional mapping, in vitro permeation testing, generalized additive modeling (GAM), and attenuated total reflectance–Fourier transform infrared (ATR-FTIR) spectroscopy were combined to evaluate formulation-dependent transport across pig and mouse skin models, complemented by exploratory human-skin experiments. **Results**: Among the nanoemulsion formulations, pig skin showed the highest salbutamol permeation, with flux values reaching approximately 390 µg/cm^2^·h. Within the PHS-based system, mouse skin showed lower permeation and a stronger dependence on formulation composition than pig skin, while the simple aqueous vehicle also produced comparatively low permeation in the murine model. The aqueous-vehicle control produced substantially higher permeation than the nanoemulsions in pig skin but lower permeation in mouse skin, while receptor-phase salbutamol concentrations remained below the limit of quantification in human skin. Across both surfactant systems, the most favorable nanoemulsion-mediated permeation was generally associated with water-rich formulations containing comparatively low surfactant levels, whereas highly surfactant-rich regions showed reduced flux despite marked lipid- or protein-associated spectral changes in the descriptive ATR-FTIR analysis. Regression analyses suggested that droplet size and viscosity alone could not consistently explain permeation behavior, whereas compositional modeling revealed pronounced non-linear effects of the water–surfactant–oil balance. **Conclusions**: Overall, this study demonstrates that nanoemulsion-mediated delivery of hydrophilic drugs is governed primarily by ternary composition, with formulation effects varying across skin models. These findings highlight the importance of composition-based formulation design and appropriate skin-model selection during the development of transdermal systems for hydrophilic drugs.

## 1. Introduction

Transdermal drug delivery (TDD) offers a non-invasive route for systemic drug administration, with potential advantages including avoidance of hepatic first-pass metabolism, reduced fluctuations in plasma drug concentration, and improved patient compliance compared with repeated oral or parenteral dosing [1,2,3]. However, the applicability of this route remains strongly limited by the barrier function of the stratum corneum (SC), particularly for hydrophilic active pharmaceutical ingredients (APIs). The SC consists of densely packed corneocytes embedded within an organized lipid-rich extracellular matrix and therefore acts as the principal barrier against molecular permeation [4,5]. Because polar molecules generally exhibit poor partitioning into this lipophilic barrier, their passive transdermal transport remains especially challenging [6,7,8].

To overcome this limitation, several formulation-based and device-assisted enhancement strategies have been investigated, including chemical penetration enhancers, vesicular carriers, energy-assisted approaches, and physical barrier-modification systems such as microneedles [5]. Among formulation-based approaches, nanoemulsions (NEs) are attractive carrier systems because they combine drug solubilization, skin hydration, large interfacial surface area, and scalable preparation methods within a single vehicle platform [9,10]. In addition, NEs can be prepared using relatively simple low-energy methods, including the phase inversion temperature (PIT) technique, which offers practical advantages in terms of manufacturing simplicity and scalability.

Although NEs are frequently associated with improved permeation performance, it remains difficult to predict how changes in NE composition influence transdermal delivery outcomes, particularly for hydrophilic APIs. Droplet size and viscosity are well-established and important physicochemical descriptors in nanoemulsion characterization, and both have been widely investigated in relation to transdermal delivery performance [1,10]. However, in multicomponent systems such as NEs, these properties are strongly influenced by the underlying balance between water, surfactant, and oil phases. Consequently, alongside conventional physicochemical characterization, a more systematic evaluation of ternary composition is needed to better understand how formulation design governs drug release and permeation behavior.

Salbutamol is a selective β_2_-adrenergic receptor agonist widely used in the treatment of asthma and other obstructive pulmonary diseases. Although inhalation remains the primary administration route, inhaled therapy is associated with technique-dependent variability and inconsistent pulmonary deposition, while oral administration suffers from low bioavailability due to extensive first-pass metabolism [11]. With a relatively low molecular weight of approximately 288 Da but high hydrophilicity (logP ≈ 0.1), salbutamol represents a clinically relevant and mechanistically useful model compound for investigating the limitations of NE-mediated transdermal delivery of hydrophilic APIs [11,12]. Successful transdermal administration of salbutamol could potentially provide sustained systemic delivery while reducing dosing frequency and improving patient convenience, particularly in populations where inhalation technique or repeated dosing may be problematic.

In our previous work, the same PIT-based formulation platform was used to investigate the influence of API hydrophilicity by comparing salbutamol and ibuprofen in polyoxyl castor oil (PCO; Kolliphor^®^ EL)-based NEs using mouse skin [13]. The present study addresses a distinct formulation question. By retaining the previously characterized PCO-based system as a controlled reference and introducing polyoxyl hydroxystearate (PHS; Kolliphor^®^ HS 15) as a structurally distinct second surfactant system, the study examines whether composition-dependent salbutamol delivery is governed consistently across surfactant architectures. The use of a related formulation framework was therefore intentional, as it enabled the effects of surfactant architecture and the ternary water–surfactant–oil balance to be evaluated without introducing additional changes to the remaining formulation platform.

The present study further extends the biological scope by comparing mouse and pig skin models. Mouse skin was included as a common permeable preclinical model, while pig skin was selected as a more structurally relevant surrogate for human skin because of its closer barrier organization and lipid composition compared with rodent skin [14,15]. Evaluating related formulation spaces across these biologically distinct barriers allowed us to determine whether composition–performance relationships, rather than only absolute flux values, were transferable between skin models. Exploratory human-skin experiments were additionally included to provide a translational reference for the passive delivery observed in the animal models. The contribution of the present work lies in integrating ternary compositional mapping and non-linear modeling with comparative permeation and complementary ATR-FTIR evaluation across two surfactant architectures and biologically distinct skin models. This integrated design allows formulation performance to be interpreted within a drug–vehicle–skin framework and the respective contributions of ternary composition, conventional physicochemical descriptors, and formulation-induced skin responses to be distinguished—an analysis that single-surfactant, single-model, permeation-only studies cannot provide.

Accordingly, the present study aimed to determine how ternary nanoemulsion composition and surfactant architecture jointly influence salbutamol permeation and how these relationships depend on the selected skin model. NEs were prepared using the low-energy PIT method, and ternary compositional mapping was combined with physicochemical characterization, in vitro permeation testing, descriptor-based regression, generalized additive modeling (GAM), and complementary ATR-FTIR analysis.

## 2. Materials and Methods

### 2.1. Materials

Salbutamol hemisulphate (>98.0% purity) was purchased from TCI Chemicals (Tokyo, Japan). The oil phase used in all formulations was medium-chain triglycerides (MCT; Miglyol^®^ 812, Ph. Eur. grade, supplied by Caelo, Hilden, Germany). Kolliphor^®^ EL (Ph. Eur. Macrogolglycerol Ricinoleate/USP–NF Polyoxyl 35 Castor Oil) and Kolliphor^®^ HS 15 (Ph. Eur. Macrogol 15 Hydroxystearate/USP-NF Polyoxyl 15 Hydroxystearate), used as surfactants, were generously provided by BASF (Ludwigshafen, Germany). Soybean-derived lecithin (≥97% purity) was obtained from Carl Roth (Karlsruhe, Germany). Pig ears were supplied by a local butcher in Bonn, Germany. Mice (C57BL/6J strain) were kindly provided by the DZNE (German Center for Neurodegenerative Diseases, Bonn, Germany) through a collaborating research group working on brain studies. All chemicals were of analytical grade.

### 2.2. Preparation of Nanoemulsions

NEs were prepared using a modified PIT method adapted from Lamprecht et al. and our previously published formulation protocol [13,16]. Briefly, formulations were prepared as 5 g batches containing fixed amounts of salbutamol hemisulphate (2.5% *w*/*w*), soy lecithin (1% *w*/*w*), and sodium chloride (NaCl) (0.9% *w*/*w*), while the relative proportions of water, surfactant, and MCT were varied according to predefined ternary compositions.

In the present study, two non-ionic surfactant systems, PCO and PHS, were investigated comparatively across 36 ternary compositions (Table S1). The aqueous phase consisted of salbutamol hemisulfate and NaCl dissolved in Milli-Q water, whereas the oil phase contained MCT, soy lecithin, and the respective surfactant. Soy lecithin was included as a phospholipid co-surfactant to support interfacial film formation and droplet stabilization, whereas NaCl was incorporated as an electrolyte to modulate the hydration of the polyoxyethylene chains of the non-ionic surfactants and thereby influence phase-inversion behavior during the PIT process. Both excipients were maintained at fixed concentrations across all formulations to isolate the effects of the water–surfactant–oil balance. The aqueous phase was added dropwise to the oil phase under continuous stirring, followed by heating to 80 °C to induce phase inversion and subsequent cooling to room temperature under continuous mixing. The formulations were prepared as two distinct nanoemulsion sets, namely polyoxyl castor oil-based nanoemulsions (PCO-NEs) using PCO and polyoxyl hydroxystearate-based nanoemulsions (PHS-NEs) using PHS as surfactants. All formulations were equilibrated overnight at 4 °C prior to characterization and permeation studies. Following overnight equilibration, the formulations were visually inspected for macroscopic phase separation. Formulations exhibiting visible phase separation were classified as physically unstable and excluded from subsequent analyses. Because the present study was designed as a systematic comparison across a broad ternary formulation space rather than the pharmaceutical qualification of a single optimized dosage form, the physicochemical characterization strategy focused on measurements that could be applied consistently across the design space.

### 2.3. Size Determinations

Particle size and polydispersity index (PDI) measurements were performed using photon correlation spectroscopy (PCS) with a Horiba Scientific nanoPartica SZ-100 system (Horiba Scientific, Kyoto, Japan), following the procedure described previously by our group [13]. Measurements were carried out at 25 °C and a detection angle of 90°. Prior to analysis, samples were diluted with Milli-Q water as required to achieve suitable scattering intensity and to minimize multiple scattering effects. All measurements were performed in triplicate, and the mean hydrodynamic diameter (Z-average) and PDI values were recorded. These measurements were used to compare the hydrodynamic dimensions and distribution width of the dispersed populations across the ternary formulation space.

Zeta potential was not included as a primary stability descriptor because both formulation systems were stabilized by non-ionic surfactants, for which stabilization is predominantly steric rather than electrostatic [17]. Moreover, electrophoretic-mobility measurements are strongly influenced by dispersant composition, ionic strength, conductivity, viscosity, and dilution conditions. Several viscous or gel-like formulations would have required substantial modification of the sample conditions to meet instrumental requirements, and the resulting values would therefore not have been directly representative of the concentrated formulations used in the permeation experiments [18,19].

### 2.4. Viscosity Determinations

Viscosity measurements were performed using a modular compact rheometer (MCR 92, Anton Paar GmbH, Graz, Austria) equipped with a cone–plate geometry (CP50-1, 50 mm diameter, 1° cone angle), following our previously reported protocol [13]. Measurements were conducted at 35 ± 0.1 °C using a Peltier temperature-control system. Approximately 0.5 mL of each formulation was equilibrated for 3 min prior to analysis.

Steady-shear measurements were carried out over a shear rate range of 1–100 s^−1^. For comparative evaluation across the ternary compositional space, effective viscosity values were calculated from the linear regression of shear stress versus shear rate using RheoCompass software 1.30 (Anton Paar GmbH, Graz, Austria). These measurements were used to compare composition-dependent differences in bulk flow behavior across the ternary formulation space.

### 2.5. In Vitro Drug Permeation Studies

In vitro permeation studies were performed using pig, mouse, and exploratory human skin models. Porcine skin was excised from pig ears and dermatomed to a thickness of 1 mm using an Aesculap ACCULAN GA 643 dermatome (Aesculap AG, Tuttlingen, Germany). Circular skin sections (18 mm diameter) were prepared using a biopsy punch. Full-thickness mouse skin was carefully excised using a scalpel, and residual hair was removed using an electric shaver prior to preparation for Franz diffusion cell experiments. Human skin samples were obtained from abdominal tissue of five different donors from plastic surgery. Ethical approval was granted by the ethics council of the University of Bonn under reference 082/20. Excess subcutaneous fatty tissue was carefully removed prior to storage, and the cleaned skin samples were frozen until further use. Before the permeation experiments, the tissues were thawed and dermatomed to a thickness of approximately 300 µm. The prepared skin was subsequently sectioned into pieces of approximately 2 cm^2^ for Franz diffusion cell studies. All skin samples were stored at −20 °C and thawed for 30 min prior to use. Before experimentation, the integrity of each skin sample was visually inspected.

Permeation experiments were conducted using Franz diffusion cells (SES Analysensysteme, Bechenheim, Germany) with a diffusion area of 1.04 cm^2^ and a receptor volume of 8 mL. All skin models were evaluated under identical Franz diffusion cell conditions to enable direct comparison between permeation datasets. The receptor chamber was filled with phosphate-buffered saline (PBS, pH 7.4), which was continuously stirred at 250 rpm using magnetic stir bars to maintain receptor-phase homogeneity throughout the experiment. The receptor compartment was maintained at 37 °C, consistent with the protocol used in our previous study [13], and the system was equilibrated for 30 min prior to formulation application. Subsequently, 500 mg of nanoemulsion formulation was applied to the donor chamber. Low-viscosity formulations were transferred using a pipette, whereas more viscous formulations were applied using a syringe to accommodate differences in flow behavior and ensure reproducible transfer. The donor compartments were closed with silicone caps and sealed with parafilm to minimize evaporation and were additionally wrapped with aluminum foil as a precaution against potential light-induced degradation of salbutamol. Aluminum foil was applied to the donor compartments only, as the receptor chambers were housed within a separate, opaque, temperature-controlled compartment of the Franz cell device and were therefore shielded from ambient light.

Receptor phase samples were collected at 0, 1, 2, 4, 6, 8, and 24 h, with fresh PBS added after each sampling step to maintain sink conditions. All permeation studies were performed in triplicate.

### 2.6. Aqueous-Vehicle Control Permeation Study

To distinguish the contribution of the aqueous vehicle from that of the complete nanoemulsion system, additional permeation experiments were performed using a simple aqueous salbutamol vehicle. Salbutamol hemisulfate (2.5% *w*/*w*, matched to the overall drug loading of the nanoemulsion formulations) and NaCl (0.9% *w*/*w*) were dissolved in Milli-Q water without MCT, surfactant, or lecithin. Complete dissolution was confirmed visually before application. Permeation was evaluated using porcine skin (*n* = 5), C57BL/6J mouse skin (*n* = 5), and seven human skin sections obtained from three donors, with skin preparation and Franz-cell conditions corresponding to those described in Section 2.5. Salbutamol was quantified by HPLC, as described in Section 2.7. Steady-state flux and cumulative permeation after 24 h were calculated using the same procedures as those applied to the corresponding NE experiments.

### 2.7. High-Performance Liquid Chromatography (HPLC) of Salbutamol

Quantitative analysis of salbutamol was performed using a previously validated HPLC method reported in our earlier study [13], with minor modifications. Analyses were carried out using a Shimadzu Prominence-I LC-2030C Plus (Shimadzu Corporation, Kyoto, Japan) HPLC system equipped with a photodiode array (PDA) detector and a reversed-phase LiChrospher^®^ 100 RP-18 column (5 µm, 250 mm × 4.6 mm; Merck KGaA, Darmstadt, Germany). The mobile phase consisted of phosphate buffer–acetonitrile (80:20, *v*/*v*), delivered isocratically at 1.0 mL/min, with detection performed at 276 nm and the column maintained at 40 °C. For the present study, calibration curves were established in the range of 0.25–50 µg/mL (R^2^ > 0.999). The calculated limit of detection (LOD) and quantification (LOQ) values were 0.30 µg/mL and 0.91 µg/mL, respectively.

### 2.8. Attenuated Total Reflectance–Fourier Transform Infrared (ATR-FTIR) Spectroscopy

ATR-FTIR measurements were performed using a PerkinElmer Spectrum Two FTIR spectrometer equipped with a single-reflection universal attenuated total reflectance (UATR) accessory fitted with a diamond crystal (PerkinElmer Inc., Waltham, MA, USA). Spectra were acquired directly from the permeation-exposed skin regions over a range of 4000–600 cm^−1^ with a spectral resolution of 4 cm^−1^ and 32 accumulated scans per spectrum, using Spectrum IR software (version 10.7.2, PerkinElmer Inc., Shelton, CT, USA, 2020). For the animal-skin experiments, three to six individual permeation-exposed skin sections were analyzed per experimental condition, depending on the number of suitable sections available following the permeation studies. Three spectra were recorded from separate locations within the exposed area of each skin section and averaged to obtain a single section-level value. Because multiple skin sections could originate from the same source animal, these measurements were not considered independent biological replicates and were evaluated descriptively as exploratory observations of local formulation–skin responses. Following the acquisition, spectra recorded in transmittance (%) mode were converted to absorbance and subjected to baseline correction, followed by spectral smoothing using a smoothing factor of 20. Processed spectra were subsequently analyzed using OriginPro 2026 software (OriginLab Corporation, Northampton, MA, USA).

Formulations F9, F19, and F21, corresponding to water/surfactant/MCT ratios of 70:10:20, 30:40:30, and 10:60:30, respectively, were analyzed in pig and mouse skin. Skin samples were obtained following the 24 h permeation experiments described in Section 2.5. Each section had been mounted separately in an individual Franz diffusion cell and exposed to its assigned treatment during the permeation experiment.

After completion of the permeation studies, the skin samples were carefully removed from the Franz diffusion cells and washed three times with PBS to remove residual formulation from the surface. Species-specific drying protocols were optimized to minimize structural artifacts. Porcine skin samples were dried for 1 h under ambient conditions in a fume hood, whereas murine skin samples were dried overnight at 22 °C before spectroscopic analysis.

In addition to nanoemulsion-treated samples, excipient-only reference experiments were performed to contextualize the contribution of individual formulation components to the ATR-FTIR response. Separate pig and mouse skin samples were exposed to aqueous phase containing 0.9% *w*/*w* NaCl, MCT, PCO, or PHS, followed by PBS washing and drying according to the procedures described above prior to ATR-FTIR analysis. The corresponding descriptive data are provided in Table S4.

Additionally, exploratory ATR-FTIR analysis was performed on human skin samples obtained from selected NE permeation experiments to provide a translational reference. For human skin, a broader set of selected formulations (F9, F11, F13, F15, F21, F24, F26, F27) was screened by ATR-FTIR as an exploratory survey of compositional variability across the ternary space.

Peak-position data were excluded from quantitative analysis because species-specific drying protocols and hydration states could affect absolute band positions. Quantitative evaluation focused on four FTIR-derived parameters: the A(CH_2_)/A(CH_3_) ratio, the A(CH)/A(Amide II) ratio, and the individual lipid-associated absorbance areas A(CH_2_) and A(CH_3_). These parameters were summarized descriptively, as described in Section 2.9.

### 2.9. Statistics

Statistical analyses were performed using RStudio version 2025.05.1 (RStudio PBC, Boston, MA, USA) and OriginPro 2026 version 10.3.0.197 (OriginLab Corporation, Northampton, MA, USA). To enable statistically valid analysis of the ternary nanoemulsion compositions, the formulation variables (water, surfactant, and MCT) were transformed into isometric log-ratio (ilr) coordinates prior to statistical modeling.

The relationships between nanoemulsion composition and transdermal flux were evaluated using Generalized Additive Models (GAMs), allowing the assessment of non-linear and interactive relationships between formulation components and permeation behavior. Partial dependence plots (PDPs) derived from the GAM analyses were additionally used to visualize the individual contributions of water, surfactant, and MCT ratios to permeation behavior while accounting for the remaining compositional variables. Droplet size and viscosity were additionally included as covariates in the regression models to investigate their potential contribution to permeation behavior. Model significance was evaluated using F-statistics and corresponding *p*-values obtained within the GAM framework. Correlation analyses between formulation parameters and flux values were additionally performed using Pearson correlation analysis in OriginPro.

ATR-FTIR data from pig and mouse skin were evaluated descriptively because multiple skin sections could originate from the same source animal and the study was not designed to provide independent animal-level replication for each treatment condition. For each skin section, the three spectra recorded within the permeation-exposed area were averaged to obtain one section-level value. The resulting values were summarized to illustrate exploratory skin-model- and treatment-associated spectral patterns but were not used for confirmatory formulation-level inference. Human-skin and excipient-only ATR-FTIR experiments were likewise evaluated descriptively.

## 3. Results

### 3.1. Nanoemulsion Preparation and Evaluation

**Figure 1:**
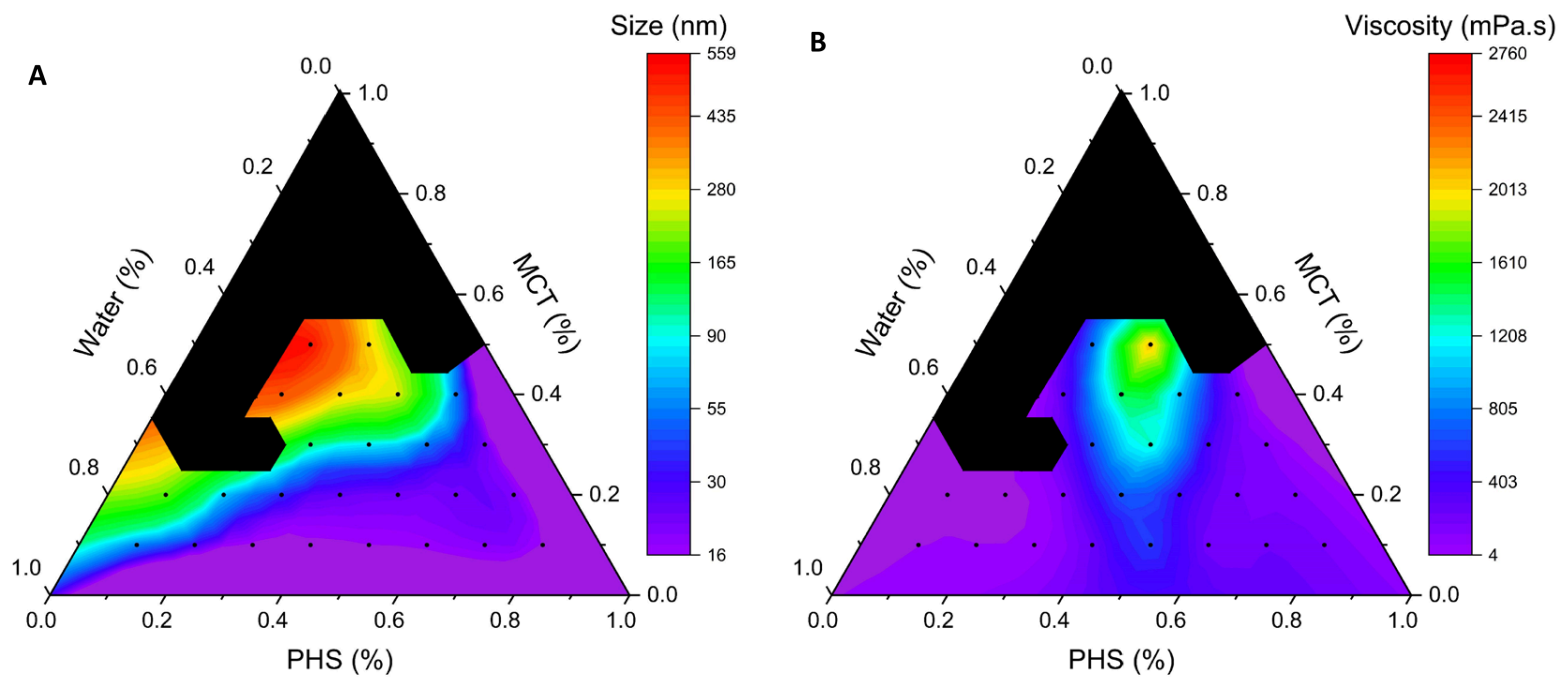
Ternary of PHS-NEs representing mean droplet size (nm) (**A**) and viscosity (mPa·s) (**B**). Each data point represents the mean of three replicate measurements (*n* = 3). Black regions indicate formulations that exhibited phase separation and were therefore considered unstable and excluded from further evaluation.

The ternary diagrams in Figure 1 illustrate the compositional behavior of PHS-NEs in terms of droplet size and viscosity. Detailed compositional analyses of PCO-NEs, including droplet size and viscosity distributions across the ternary composition space, have been reported previously using the same formulation library [13]. Black regions correspond to formulations that exhibited phase separation following overnight storage at +4 °C and were excluded from subsequent analyses. Among the 36 formulations prepared, eleven PHS-NEs were identified as unstable.

As shown in Figure 1A, PHS successfully generated nanoemulsions with droplet sizes ranging from 17 nm to 559 nm, with only one formulation exceeding 500 nm. Furthermore, 19 of the 25 stable PHS-NEs exhibited droplet sizes below 80 nm, indicating the formation of generally fine nanoemulsion systems. The smallest droplets were generally observed in the surfactant-rich corner of the ternary diagram.

Figure 1B illustrates the viscosity distribution of the PHS-NEs, which ranged from 3.6 to 2752 mPa·s. Most formulations remained relatively fluid, particularly in the water-rich region of the ternary space. Elevated viscosity values were observed in a limited number of formulations with lower water contents and higher combined PHS and MCT fractions. Thus, increased viscosity was restricted to a small compositional region rather than being representative of the PHS-NE system as a whole.

### 3.2. Skin Permeation Behavior of the Drug

The transdermal permeation behavior of salbutamol was evaluated using nanoemulsion formulations that met the preliminary physical stability criterion, defined as the absence of visible phase separation following overnight storage at 4 °C.

#### 3.2.1. Permeation Behavior of the Drug Through Pig Skin

**Figure 2:**
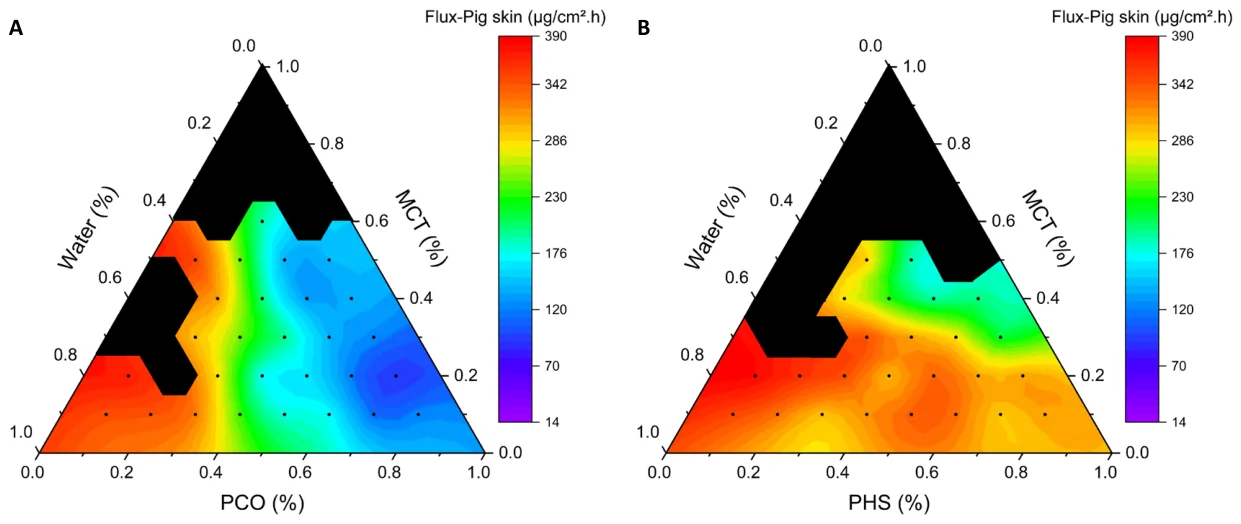
Ternary diagrams representing the steady-state flux (µg/cm^2^·h) of Salbutamol through pig skin treated with PCO-NEs (**A**) and PHS-NEs (**B**). The color scale indicates the magnitude of drug permeation. The black areas indicate compositions characterized by phase separation, which were excluded from the permeation studies. Each data point represents the mean of three independent permeation experiments (*n* = 3).

Permeation studies were performed using 28 newly prepared PCO-NEs corresponding to compositions previously identified as physically stable [13], as well as the 25 PHS-NEs that met the preliminary physical stability criterion described above. The newly prepared PCO-NEs were recharacterized before permeation testing, and their droplet size and PDI values were consistent with those reported previously [13]. The resulting steady-state flux values demonstrated a strong dependence on both surfactant type and formulation composition, as illustrated in the ternary flux diagrams (Figure 2).

For the PCO-NEs, salbutamol flux values through pig skin ranged from 89 to 381 µg/cm^2^·h. The highest permeation was obtained with the formulation containing 70% water, 10% PCO, and 20% MCT. Overall, the highest flux regions obtained from PCO-NEs were predominantly associated with water-rich formulations containing comparatively low surfactant ratios.

On the other hand, salbutamol flux values ranged from 177 to 390 µg/cm^2^·h with the PHS-NEs with the highest permeation again observed with the formulation containing 70% water, 10% PHS, and 20% MCT. However, unlike PCO-NEs, several intermediate-compositional PHS-NEs containing more balanced water/PHS/MCT ratios (like the formulation containing 40% water, 30% PHS, and 30% MCT showed 370 µg/cm^2^·h of flux) also maintained relatively high permeation performance, indicating a broader high-flux compositional region across the ternary diagram.

To further investigate the influence of NE composition on transdermal delivery performance, the individual effects of water, surfactant, and MCT ratios on salbutamol flux were analyzed using PDPs derived from the GAM. This approach enabled the evaluation of how compositional changes influenced permeation behavior across both surfactant systems (PCO and PHS). By varying one compositional parameter while holding the remaining variables constant, the PDPs provided a detailed visualization of the independent contribution of each NE component to the observed flux behavior.

**Figure 3:**
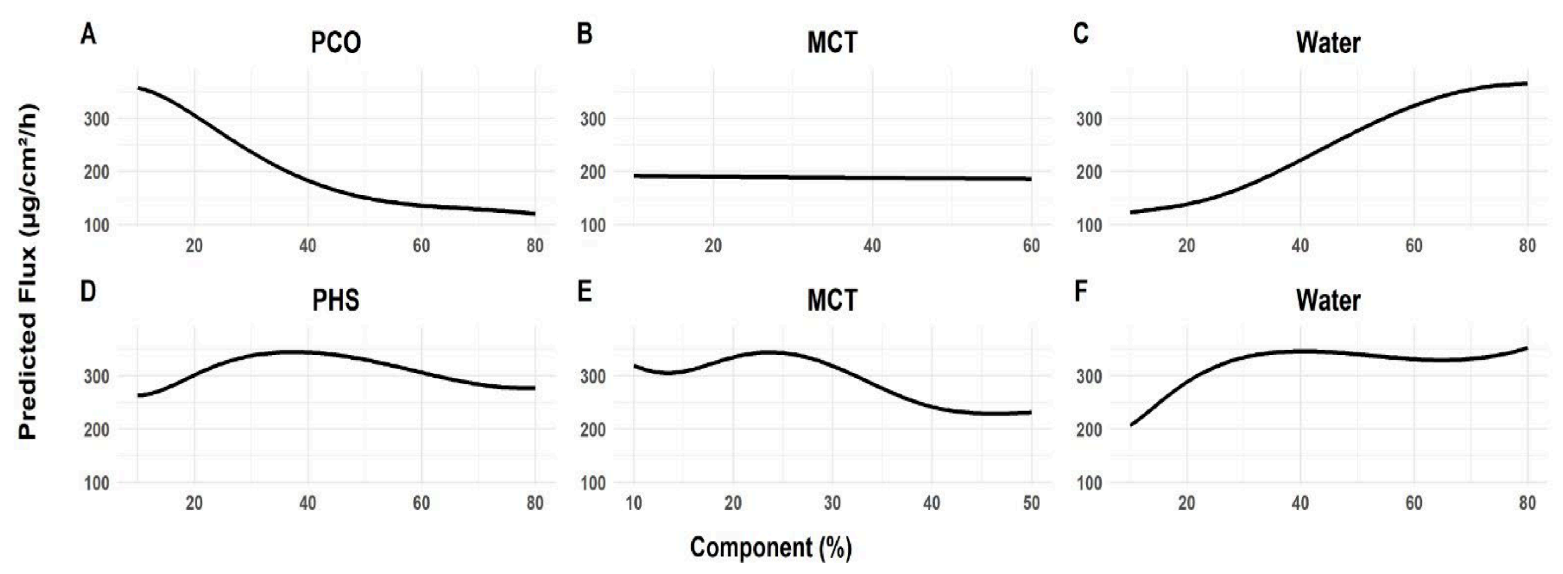
Partial dependence plots (PDPs) illustrating the individual non-linear effects of ternary nanoemulsion components on steady-state salbutamol flux (µg/cm^2^·h) through porcine skin. The upper panels represent PCO-NEs plotted as a function of (**A**) PCO, (**B**) MCT, and (**C**) Water. The lower panels represent PHS-NEs plotted as a function of (**D**) PHS, (**E**) MCT, and (**F**) Water. Each curve visualizes the predicted flux response modeled by the Generalized Additive Model (GAM) for a single component while holding the remaining two formulation variables constant at their respective mean values. Each formulation-level flux value used for modeling represents the mean of three independent permeation experiments (*n* = 3); the models included 28 PCO-NE and 25 PHS-NE formulations.

For the pig skin dataset with the PCO-NEs (Figure 3), PCO demonstrated a strong non-linear negative relationship with flux, with predicted permeation declining steeply as surfactant concentration increased, indicating that excessive surfactant suppresses transdermal delivery. MCT showed the most stable trend among the three components, displaying a relatively linear and slightly negative association with flux. In contrast, water exhibited a clear positive association with flux, although the relationship was distinctly non-linear. Increasing water content generally enhanced permeation, although fluctuations were observed across the curve.

In contrast, the PHS-NEs displayed different compositional dependencies. PHS concentration exhibited a strong non-linear, bell-shaped relationship with flux, reaching a maximum at intermediate surfactant levels (~40%) before declining at both lower and higher PHS fractions. This inverted U-shape differed markedly from the monotonic negative trend observed for PCO, indicating the presence of an optimal surfactant concentration range for permeation. MCT also displayed a pronounced bell-shaped relationship, with flux peaking at approximately 25–30% oil content before declining at both lower and higher MCT proportions. Water content showed a strong positive but non-linear effect, with flux increasing steeply at lower water fractions and approaching a plateau at higher water contents (>60%). Overall, the PDPs demonstrated that all three formulation components influenced permeation in a distinctly non-linear manner.

GAM analyses demonstrated strong non-linear relationships between NE composition and salbutamol flux in the pig skin datasets. The PCO-NEs model showed particularly high explanatory performance (adjusted R^2^ = 0.903), whereas the PHS-NEs exhibited a comparatively lower, yet still substantial, model fit (adjusted R^2^ = 0.686). These findings indicate that flux behavior in the PCO-NEs followed more predictable compositional trends, while the PHS-NEs displayed greater composition-dependent variability in permeation behavior.

In addition to compositional analyses, regression analyses were performed to investigate whether the observed permeation behavior could be directly associated with physicochemical properties such as droplet size and viscosity. Investigating the pig skin dataset treated with PCO-NEs, the regression model demonstrated only a weak association between physicochemical parameters and permeation behavior (R^2^ = 0.044), indicating that only 4.4% of the variance in flux was explained by these two predictors. Neither droplet size (*p* = 0.418) nor viscosity (*p* = 0.442) emerged as significant predictors of flux, indicating that the permeation behavior of the PCO-NEs through pig skin could not be sufficiently explained by these parameters alone.

For the pig-skin dataset treated with PHS-NEs, the regression model exhibited moderate explanatory power (R^2^ = 0.269). Viscosity showed a significant negative association with flux (*p* = 0.028) while droplet size remained non-significant (*p* = 0.445). These findings suggest that viscosity contributed partially to flux variability between the PHS-NEs, although compositional effects remained dominant.

#### 3.2.2. Permeation Behavior of the Drug Through Mouse Skin

**Figure 4:**
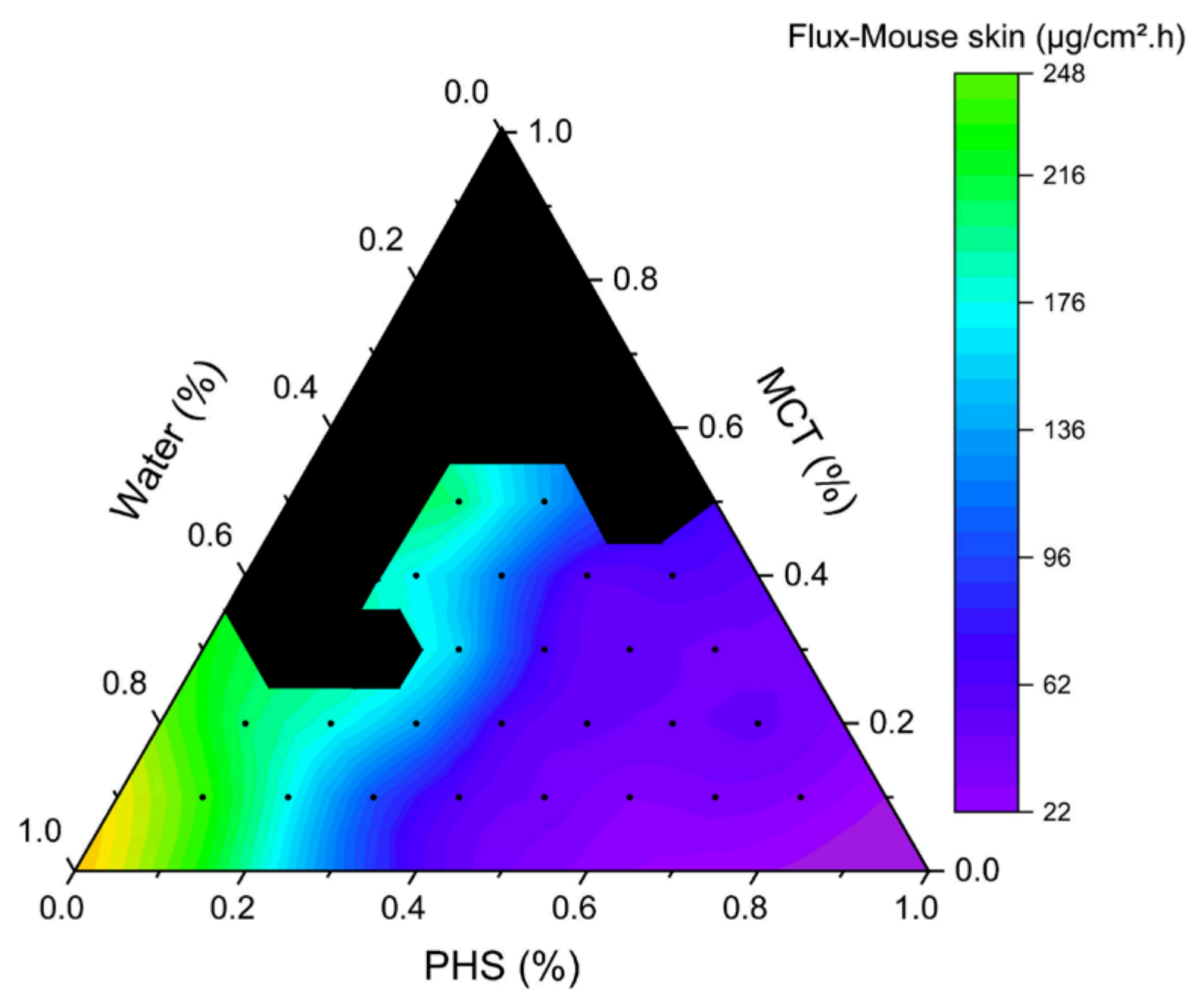
Ternary diagrams representing the steady-state flux (µg/cm^2^·h) of salbutamol through mouse skin treated with PHS-NEs. The color scale indicates the magnitude of drug permeation. The black areas indicate compositions characterized by phase separation, which were excluded from the permeation studies. Each data point represents the mean of three independent permeation experiments (*n* = 3).

Mouse-skin permeation experiments in the present study were performed using the 25 stable PHS-NEs, and the resulting steady-state flux values are presented in Figure 4. Detailed compositional and permeation analyses of the corresponding PCO-based formulation set in mouse skin were reported previously [13]. Those data are therefore not reproduced here and are used only as an explicitly cited contextual reference for cross-surfactant and cross-model interpretation.

Flux values for PHS-NEs ranged from 25 to 242 µg/cm^2^·h. The highest permeation was obtained with the formulation containing 80% water, 10% PHS, and 10% MCT. However, several formulations containing intermediate water ratios and elevated MCT contents also maintained relatively high permeation performance. The most consistent compositional trend observed in the mouse skin datasets obtained from PHS-NEs was the reduction in flux at higher surfactant concentration.

The GAM approach was subsequently applied to the mouse skin treated with PHS-NEs dataset to evaluate the compositional effects governing salbutamol permeation in the murine model. The resulting model yielded an adjusted R^2^ of 0.81, indicating a strong relationship between formulation composition and permeation behavior.

**Figure 5:**
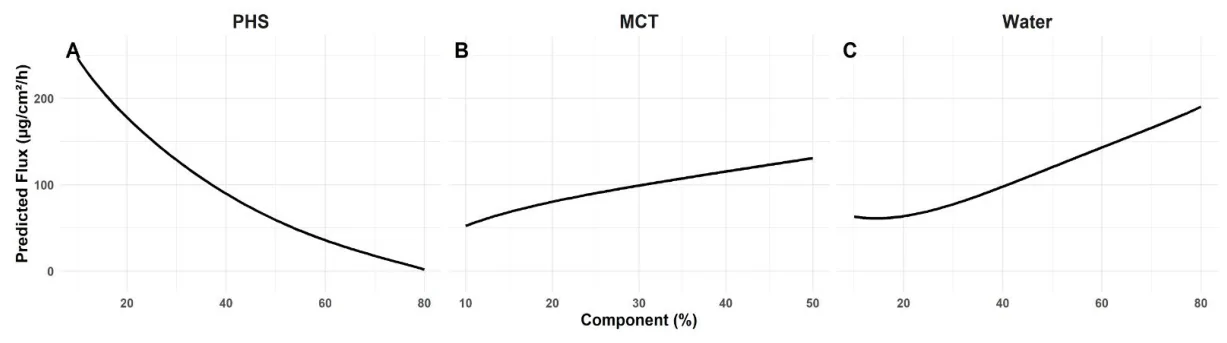
Partial dependence plots of PHS-NEs on mouse skin permeation. These plots represent the individual impact of (**A**) PHS, (**B**) MCT, and (**C**) Water on predicted salbutamol flux through the murine barrier. Each formulation-level flux value used for modeling represents the mean of three independent permeation experiments (*n* = 3); the model included 25 PHS-NE formulations.

For the mouse PHS-NEs, PHS concentration showed a marked negative relationship with salbutamol flux (Figure 5A), with elevated surfactant concentrations associated with reduced permeation across the murine barrier. The MCT-dependent PDP showed a positive relationship between oil content and predicted flux within the investigated formulation space (Figure 5B). Water content showed the strongest positive compositional trend in the present PHS-based mouse dataset (Figure 5C), with a slight initial decline at very low ratios followed by a pronounced increase in predicted flux above approximately 20% water. Thus, both water and MCT contributed positively to the modeled permeation behavior, while their relative influence should be interpreted specifically within the PHS-based murine formulation system.

Regression analyses were further performed to assess whether droplet size and viscosity could explain salbutamol permeation through mouse skin. For the PHS-NEs, the model showed a moderate association between physicochemical parameters and flux (R^2^ = 0.248). Droplet size was significantly and positively associated with salbutamol flux (*p* = 0.014), whereas viscosity was not a significant predictor (*p* = 0.310).

#### 3.2.3. Permeation from a Simple Aqueous Vehicle

Salbutamol permeation from the simple aqueous vehicle differed markedly among the investigated skin models. In porcine skin, the aqueous vehicle produced a mean flux of 969 ± 170 µg/cm^2^·h and a cumulative permeation of 84.5 ± 3.4% of the applied dose after 24 h. Its mean flux was approximately 2.5-fold higher than the highest mean flux obtained with the porcine-skin NE formulations, which was 390 µg/cm^2^·h for the PHS-based system. In murine skin, permeation from the aqueous vehicle was substantially lower, with a mean flux of 11.8 ± 4.6 µg/cm^2^·h and a 24 h cumulative permeation of 2.9 ± 1.0% of the applied dose. This flux was below the range obtained with the PHS-based NEs evaluated in the present study (25–242 µg/cm^2^·h) and below the range previously reported for the PCO-based salbutamol NEs in mouse skin (14–374 µg/cm^2^·h) [13]. In human skin, receptor-phase salbutamol concentrations remained below the HPLC limit of quantification throughout the 24 h experiment in all seven skin sections obtained from three donors.

### 3.3. ATR-FTIR Results

**Figure 6:**
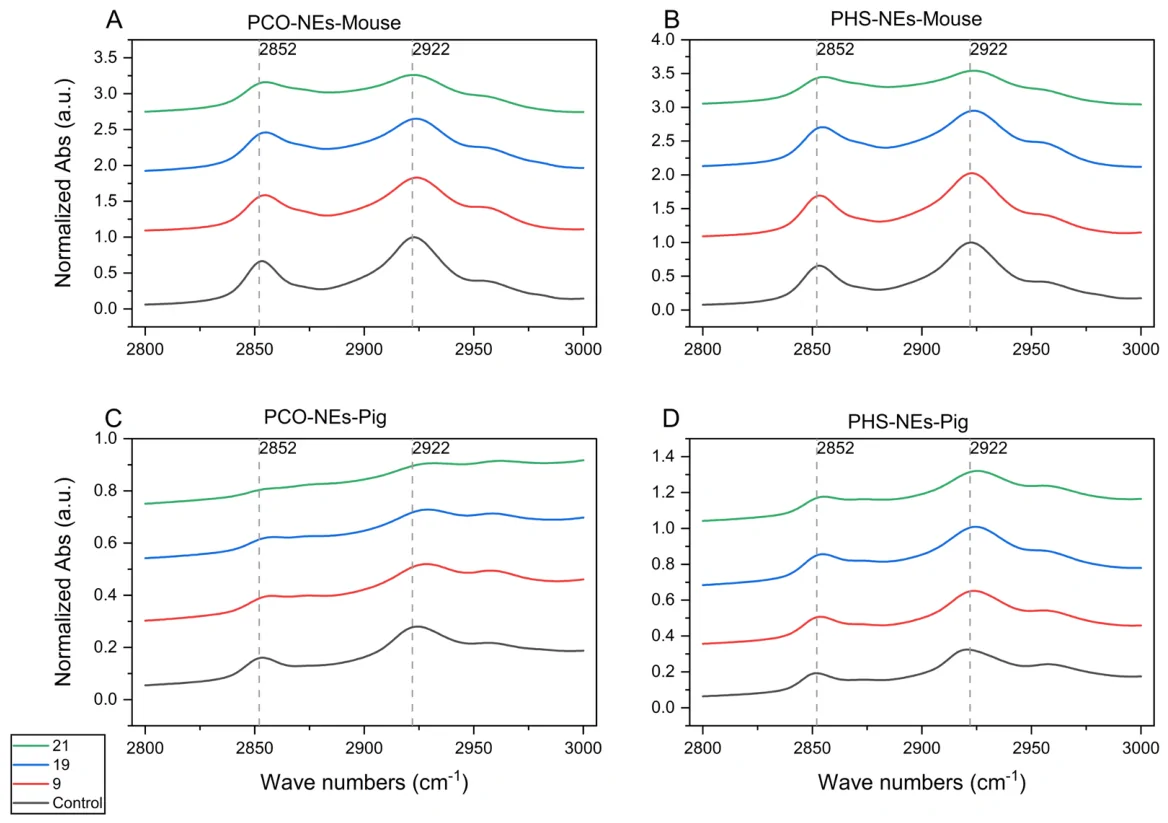
Between three and six individual permeation-exposed skin sections were available per animal-skin condition. Representative spectra are shown and were normalized and vertically offset for clarity. Three spectra recorded from separate locations within each section were averaged to obtain one section-level value for descriptive evaluation. The section-level measurements were not considered independent biological replicates because multiple sections could originate from the same source animal.

PBS-exposed control pig- and mouse-skin sections were first examined descriptively to characterize baseline spectral patterns in the two skin models. Detailed numerical datasets and additional FTIR-derived parameters are provided in Supplementary Table S2. The mean total lipid-associated area [A(CH total)] was approximately 8.3 in mouse skin and 1.6 in pig skin. The corresponding mean A(CH_2_)/A(CH_3_) ratios were 2.01 and 1.37, while the mean A(CH)/A(Amide II) ratios were 3.44 and 0.29, respectively. These descriptive baseline differences are apparent in the representative CH-stretching ATR-FTIR spectra shown in Figure 6.

**Figure 7:**
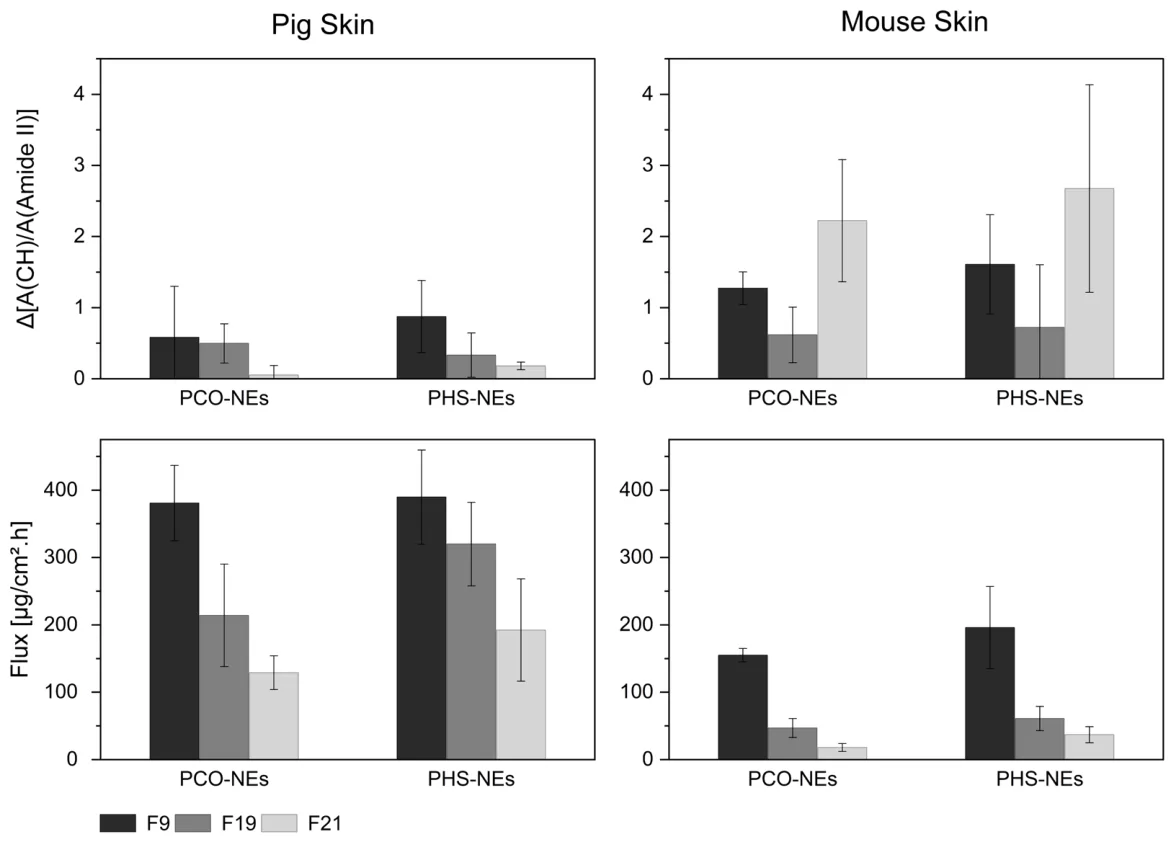
Control-normalized changes in the relative lipid-to-protein ratio Δ[A(CH)/A(Amide II)] and corresponding steady-state flux values for selected nanoemulsion formulations. Upper panels show Δ[A(CH)/A(Amide II)] values relative to PBS-exposed control skin, while lower panels show the corresponding steady-state flux values (µg/cm^2^·h). Formulations 9, 19, and 21 represent high-, intermediate-, and low-flux regions of the formulation space, respectively. Results are shown for pig skin (**left panels**) and mouse skin (**right panels**) following treatment with PCO-NEs and PHS-NEs. ATR-FTIR values are presented descriptively as mean ± SD of the available skin-section observations for each condition. The number of available sections ranged from 3 to 6 per condition; these values represent section-level observations rather than independent animals. Three spectra recorded within each permeation-exposed section were averaged to obtain one section-level value. The corresponding flux values are shown using the replicate structure of the permeation experiments.

**Figure 8:**
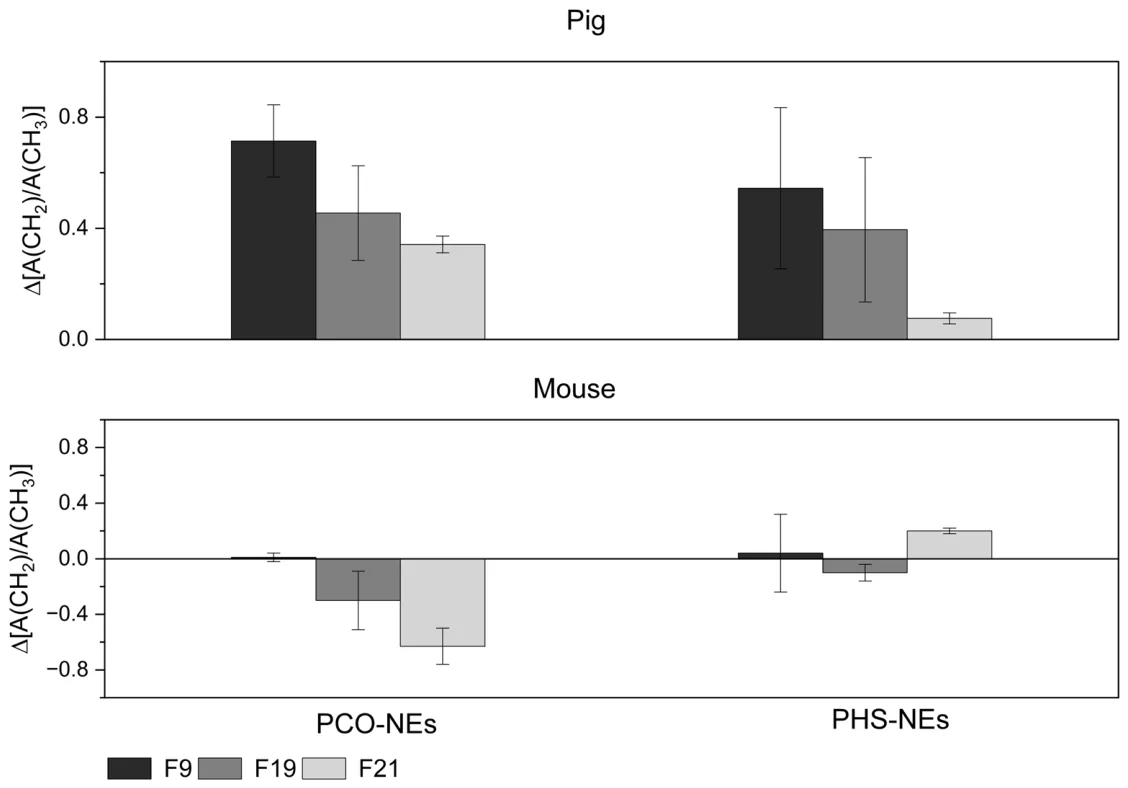
Control-normalized changes in the lipid-associated absorbance ratio Δ[A(CH_2_)/A(CH_3_)] following treatment with selected nanoemulsion formulations. Values were calculated relative to PBS-exposed control skin. Formulations 9, 19, and 21 represent distinct compositional regions of the ternary compositional map and correspond to high-, intermediate-, and low-flux formulations, respectively. Results are shown separately for pig skin (**upper panel**) and mouse skin (**lower panel**) following treatment with PCO-NEs and PHS-NEs. Data are presented descriptively as mean ± SD of the available skin-section observations for each condition. The number of available sections ranged from 3 to 6 per condition; these values represent section-level observations rather than independent animals. Three spectra recorded within each permeation-exposed section were averaged to obtain one section-level value.

Formulations 9, 19, and 21 were selected to represent high-, intermediate-, and low-flux regions of the permeation landscape while covering distinct regions of the ternary compositional space, ranging from water-rich/surfactant-poor to surfactant-rich/water-poor systems. PBS-exposed control skin served as the reference condition. Following nanoemulsion exposure, qualitative differences were observed in the lipid-associated CH-stretching region, particularly around the CH_2_ symmetric (~2852 cm^−1^) and CH_2_ asymmetric (~2922 cm^−1^) bands. The direction and magnitude of these section-level patterns differed descriptively according to skin model and surfactant system. To facilitate within-model comparison, the FTIR-derived values are subsequently expressed as control-normalized parameters in Figure 7 and Figure 8.

To minimize the influence of baseline spectral differences and facilitate comparison across skin models, further FTIR data were expressed as control-normalized Δ values.

Control-normalized Δ[A(CH)/A(Amide II)] values are shown together with the corresponding steady-state flux values in Figure 7. Descriptively, positive Δ[A(CH)/A(Amide II)] values were observed in several pig- and mouse-skin conditions following nanoemulsion exposure. In the analyzed pig-skin sections, the pattern broadly resembled the selected permeation ranking for both surfactant systems, with higher mean values for F9 than for F21. This correspondence was less consistent in mouse skin. These section-level patterns were considered exploratory and were not used to establish confirmatory formulation effects.

In the analyzed mouse-skin sections, F21 showed comparatively high Δ[A(CH)/A(Amide II)] values despite low flux. Because A(CH)/A(Amide II) is a ratio, high values may result not only from changes in lipid-associated CH absorbance but also from relatively low Amide II contributions. The supplementary descriptive data suggest that the F21 pattern was associated partly with lower Amide II values rather than proportional lipid enrichment. Accordingly, A(CH)/A(Amide II) was interpreted cautiously as a descriptive relative spectral measure and not as a direct measure of barrier disruption or permeation enhancement.

Descriptive differences were observed in the control-normalized Δ[A(CH_2_)/A(CH_3_)] values that are shown in Figure 8. In the analyzed pig-skin sections, mean values were positive across the selected formulations and both surfactant systems, with the highest descriptive values generally observed for F9. Mouse-skin sections showed a different pattern: PCO-treated sections remained close to the control value for F9 and showed lower mean values for F19 and F21, whereas PHS-treated sections exhibited smaller changes. These exploratory patterns suggest that the relative CH_2_- and CH_3_-associated spectral contributions may respond differently according to skin model and surfactant system. However, the section-level data were not interpreted as confirmatory evidence of formulation effects or as universal predictors of permeation performance.

The exploratory excipient-only measurements showed descriptive spectral patterns that differed from those observed after exposure to the complete nanoemulsions (Table S4). In mouse-skin sections, the aqueous phase and MCT were associated with relatively high A(CH)/A(Amide II) values, whereas smaller or different patterns were observed in pig-skin sections. Given the limited number of excipient-only measurements, these findings were interpreted descriptively and suggest only that the complete nanoemulsion response cannot be explained solely by the individual component measurements.

Because several analyzed skin sections originated from the same source animals, the ATR-FTIR results were interpreted descriptively rather than as independent biological replicates. Across the selected conditions, both the baseline spectral profiles and the direction and magnitude of treatment-associated section-level changes differed descriptively between pig and mouse skin. These patterns did not consistently correspond to the permeation ranking and were therefore considered exploratory supportive evidence of local formulation–skin interactions rather than confirmatory statistical or mechanistic evidence of formulation effects.

In the exploratory human-skin experiments, receptor-phase salbutamol concentrations remained below the LOQ. The corresponding ATR-FTIR data were evaluated descriptively and are presented in Table S3 and Figure S1.

## 4. Discussion

The present study employed a controlled comparative framework based on the previously characterized PIT-derived PCO salbutamol NE platform to evaluate the effects of ternary composition, surfactant architecture, and biological barrier on transdermal delivery. Retaining the PCO-based formulation platform as the reference system while introducing PHS across the same predefined ternary framework reduced formulation-level confounding and enabled assessment of whether composition–performance relationships were preserved across surfactant systems and skin models. The results indicate that salbutamol permeation was governed mainly by the ternary balance between water, surfactant, and oil, but that the manner in which this balance was translated into receptor-phase transport depended on both surfactant architecture and the selected skin model. Overall, the data support a drug–vehicle–skin framework in which vehicle composition governs drug availability and the nature of the barrier response, while neither conventional physicochemical descriptors nor the magnitude of formulation-induced spectral changes alone consistently predicted permeation performance.

Droplet size and viscosity are frequently considered major determinants of NE performance in transdermal delivery. Smaller droplets may increase interfacial surface area and contact with the SC, while lower viscosity may improve spreading and drug release [1,5,10]. Based on this conventional view, the fine droplet sizes and relatively low viscosities observed for several PHS-NEs would be expected to produce consistently superior permeation. However, the regression analyses showed that these descriptors had only context-dependent predictive value. Droplet size was associated with flux in the murine datasets, viscosity contributed only to PHS-NE performance in pig skin, and neither parameter sufficiently explained PCO-NE permeation through porcine skin. Importantly, none of the descriptor-based models explained more than approximately one-third of the flux variability, confirming that droplet size and viscosity alone cannot account for the behavior observed across the ternary formulation space.

This finding does not contradict studies linking droplet size or viscosity to transdermal delivery, but it highlights an important methodological distinction. In many reports, droplet size is modified by processing variables such as homogenization pressure, sonication time, or energy input, while formulation composition is kept relatively constant [20,21,22]. Under those conditions, droplet size can be treated more directly as an independent variable. In the present design, by contrast, droplet size and viscosity arose from systematic variation in water, surfactant, and oil ratios. They therefore acted mainly as secondary descriptors of the compositional environment that generated them. The positive size–flux association observed in mouse skin should consequently not be interpreted as evidence that larger droplets intrinsically enhance salbutamol permeation. Rather, it most likely reflects co-variation between droplet size and the underlying water–surfactant–oil balance. Similarly, although higher viscosity may restrict molecular mobility and diffusion [23,24], its effect was not universal and depended on both the surfactant system and the skin model.

Compositional modeling provided a more coherent explanation of the permeation behavior. The GAMs captured the non-linear influence of ternary composition more effectively than descriptor-based regression, with adjusted R^2^ values between 0.686 and 0.903. The PDPs further indicated that salbutamol flux was not controlled by a single component, but by non-linear interactions among water, surfactant, and MCT. Among these variables, water showed the most consistent positive contribution across the present datasets. Water-rich formulations containing comparatively low surfactant levels, particularly F9, produced favorable permeation across both surfactant systems in pig skin. In the present PHS-based mouse dataset, water showed the strongest positive trend, whereas this pattern differed in emphasis from the previously reported PCO-based murine dataset [13], in which oil-related effects were more prominent. Mechanistically, water-rich vehicles can hydrate and plasticize the SC, increasing molecular mobility within the barrier and reducing diffusional resistance [25,26,27]. This effect is especially relevant for hydrophilic molecules, whose transport is limited by the lipophilic organization of the SC [28]. In addition, because salbutamol preferentially resides in aqueous domains rather than in the oil phase, a high water fraction may maintain drug mobility within the vehicle and preserve a freely diffusible fraction at the formulation–skin interface [10,23].

In contrast, increasing surfactant concentration generally restricted salbutamol permeation, although the magnitude of this effect depended on surfactant architecture. PCO showed a predominantly inhibitory surfactant–flux relationship, consistent with the previous PCO-based murine study [13]. PHS displayed broader tolerance to surfactant content, maintaining relatively high flux at low-to-intermediate ratios before declining at higher concentrations. This difference may partly reflect the nominal HLB values and structural characteristics of the two surfactants. PCO has a nominal HLB of approximately 12–14, whereas PHS has a somewhat higher nominal HLB of approximately 14–16 and contains approximately 30% free PEG [29,30]. The comparatively higher nominal hydrophilicity and free PEG content of PHS may contribute to its broader tolerance at low-to-intermediate surfactant concentrations, potentially by supporting greater interfacial hydration [30,31]. However, nominal HLB values provide only a general indication of surfactant hydrophilicity and do not, by themselves, predict the organization or permeation behavior of a multicomponent formulation. At high surfactant levels, both systems ultimately reduced permeation. This decline is most plausibly explained by reduced thermodynamic activity rather than by insufficient solubilization. Excess surfactant can retain salbutamol within micellar, interfacial, or polyoxyethylene-rich domains, thereby lowering the freely available drug fraction for partitioning into the skin [32,33,34]. This solubility–permeability trade-off is illustrated by the surfactant-rich F21 formulation, which induced pronounced formulation–skin interaction but yielded low flux [34,35]. Thus, for hydrophilic APIs, increasing solubilization capacity does not necessarily improve delivery if drug release and interfacial availability are compromised.

The contribution of MCT depended on both skin model and surfactant architecture. In pig skin, oil content generally appeared secondary to the effects of water and surfactant concentration. In the present PHS-based mouse dataset, water showed the strongest positive trend, but MCT also contributed positively within the investigated compositional space. Oil-related effects were more prominent in the previously reported PCO-based murine dataset [13], indicating that the relative contributions of water and oil differed according to surfactant architecture. These model- and surfactant-dependent differences may reflect variation in vehicle structure, local formulation deposition, and formulation–skin contact, although the present experiments do not distinguish among these mechanisms.

The aqueous-vehicle control experiments further qualified these composition-dependent relationships by demonstrating that the contribution of water was strongly dependent on the skin model. In murine skin, the aqueous vehicle produced lower permeation than the PCO- and PHS-based NEs, showing that the positive association between water fraction and flux within the ternary formulation space does not imply that water alone can reproduce NE performance. Rather, the non-aqueous components and the resulting multicomponent vehicle structure provided an additional contribution to transport. In porcine skin, however, the aqueous vehicle produced substantially greater permeation than the NEs, suggesting that the immediate availability of freely dissolved salbutamol was a major determinant of transport. Incorporation into the multicomponent nanoemulsion vehicle, including partitioning into interfacial or polyoxyethylene-rich domains, may have reduced the freely available drug fraction relative to the simple aqueous solution, thereby limiting partitioning from the vehicle into the skin [33,34]. In human skin, receptor-phase salbutamol concentrations remained below the LOQ for both the aqueous vehicle and the NE formulations. Thus, the aqueous vehicle did not reproduce NE performance in murine skin, yet neither water nor NE structure acted as a universal permeation-enhancing mechanism across the investigated models. This model-dependent interpretation is consistent with our previous finding that water content enhanced ibuprofen permeation despite its lipophilic nature, most likely through hydration-induced modification of the SC rather than direct aqueous solubilization [13]. Taken together with our previous findings, these observations suggest that water can exert a favorable influence on permeation across drugs of different polarity, although the magnitude of this effect remains dependent on the skin model and the complete formulation environment.

The descriptive ATR-FTIR findings were broadly consistent with the interpretation that spectral changes cannot be equated directly with permeation enhancement. In the analyzed pig-skin sections, the water-rich high-flux formulations showed spectral patterns compatible with hydration-associated stratum-corneum responses. Such patterns may be consistent with corneocyte swelling or changes in intercellular hydration, although the present measurements do not establish these mechanisms directly [25,36,37]. In the analyzed mouse-skin sections, the surfactant-rich F21 formulation showed marked variability in A(CH)/A(Amide II) despite low salbutamol flux. Because this ratio was influenced by the Amide II denominator, it was interpreted cautiously and did not provide confirmatory evidence of protein-associated barrier perturbation. Overall, the descriptive findings suggest that the presence or magnitude of a spectral change alone is insufficient to explain the delivery of a hydrophilic drug when the vehicle may simultaneously limit drug availability.

The descriptive FTIR patterns differed between the analyzed pig- and mouse-skin sections, consistent with known structural and biochemical differences between preclinical skin models [14,15,25]. However, the two skin models underwent different post-permeation drying protocols because of their distinct drying behavior. Absolute between-model spectral differences may therefore have been influenced partly by residual hydration and sample processing. All sections within each skin model underwent the same post-permeation procedure, allowing exploratory within-model comparisons, but the data were not designed for confirmatory animal-level inference. In pig skin, the descriptive lipid-associated patterns broadly resembled the selected permeation ranking, whereas in mouse skin, marked spectral changes did not consistently correspond to higher receptor-phase transport. The limited excipient-only measurements also showed patterns that differed from those observed for the complete nanoemulsions, suggesting that the complete formulation response cannot be explained solely by the individual component measurements. Accordingly, the ATR-FTIR findings should be interpreted as supportive observations of formulation–skin interactions rather than as standalone predictors of salbutamol flux, consistent with the known sensitivity of FTIR-derived parameters to hydration state, sample preparation and molecular environment rather than to drug transport alone.

A notable outcome was that salbutamol permeation did not follow the simplified permeability hierarchy often assumed for animal skin models. Rodent skin is commonly considered more permeable than porcine skin because of its thinner SC and higher follicular density [14,15]. However, in the present formulation space, pig skin supported broader high-flux regions and higher maximum steady-state flux values, particularly for water-rich, low-surfactant NEs. This suggests that permeability rankings are not fixed, but depend on the permeant and vehicle. For a highly hydrophilic molecule such as salbutamol, transport may depend more strongly on hydration-associated barrier adaptation, aqueous diffusion pathways, and the freely diffusible drug fraction at the formulation–skin interface than on SC thickness alone [37]. The follicle-rich structure of mouse skin may additionally favor local intrafollicular retention or reservoir formation without proportional enhancement of receptor-phase permeation, since follicles can function both as shunt pathways and storage sites [38,39].

Exploratory experiments with human skin provided an additional translational reference, with receptor-phase salbutamol concentrations remaining below the LOQ under the tested conditions. Although these data were not suitable for comparative modeling, they indicate that the permeation performance observed in animal skin may overestimate passive delivery across the intact human barrier.

From a translational perspective, the present design-space analysis provides a basis for selecting candidate formulations for subsequent development rather than qualifying any individual formulation for therapeutic use. The water-rich, comparatively low-surfactant region identified here represents the most relevant starting point for further formulation optimization, although favorable permeation performance alone does not establish biological safety. Once specific candidate formulations have been selected and optimized, formulation-specific dermal safety and biocompatibility should be evaluated through appropriate assessments of cytotoxicity, skin irritation and sensitization, barrier integrity and recovery, and repeated-exposure tolerability before further preclinical development.

Overall, this study supports a composition- and model-dependent view of NE-based transdermal delivery. For hydrophilic APIs, the observed performance appears to depend on sufficient aqueous drug mobility, limited surfactant-mediated retention, and skin responses compatible with polar transport rather than nonspecific perturbation. These findings emphasize that rational nanoemulsion design should move beyond isolated descriptors such as droplet size and viscosity and instead evaluate the complete drug–vehicle–skin triad.

## 5. Conclusions

This study systematically investigated how ternary nanoemulsion composition and surfactant architecture jointly influence salbutamol permeation and whether the resulting composition–performance relationships are transferable across different skin models. The results showed that permeation behavior was governed predominantly by the balance between water, surfactant, and oil, whereas conventional formulation descriptors such as droplet size and viscosity displayed only limited predictive value.

The combined permeation and complementary descriptive findings further indicated that formulation performance was determined primarily by composition and drug availability at the formulation–skin interface. Water-rich formulations generally favored salbutamol transport, while highly surfactant-rich regions tended to show reduced permeation; however, the magnitude and shape of these relationships depended on surfactant architecture and skin model. Observable spectral changes in the analyzed skin sections did not consistently correspond to permeation performance, reinforcing the idea that descriptive ATR-FTIR findings should not be interpreted as direct evidence of permeation enhancement.

The study also showed that formulation performance was strongly dependent on the selected skin model. The distinct responses observed across the porcine and murine models support the view that skin-model selection should be considered in relation to both formulation characteristics and permeant properties rather than according to fixed permeability rankings. While exploratory human skin experiments suggest important translational limitations for passive delivery, the relationships identified in this work provide a mechanistically informed framework for the rational optimization of nanoemulsion-based transdermal systems and for their future integration with complementary permeation-enhancement strategies.

## Data Availability

The data presented in this study are available from the corresponding author upon reasonable request.

## Supplementary Materials

The following supporting information can be downloaded at https://www.mdpi.com/article/10.3390/pharmaceutics18091127/s1. Table S1. Compositional design of the nanoemulsion formulations explored in this study. Table S2. Descriptive summary of ATR-FTIR-derived lipid- and protein-associated parameters following nanoemulsion treatment of pig and mouse skin. Table S3. Descriptive ATR-FTIR-derived lipid-associated parameters from exploratory human-skin experiments. Figure S1. Representative FTIR CH-stretching spectra of human skin following treatment with nanoemulsion formulations containing PCO or PHS. Table S4. Descriptive ATR-FTIR-derived lipid- and protein-associated spectral parameters from excipient-only reference experiments in pig and mouse skin.

## References

[1] Marwah H., Garg T., Goyal A.K., Rath G. (2016). Permeation Enhancer Strategies in Transdermal Drug Delivery. Drug Deliv..

[2] Jeong W.Y., Kwon M., Choi E.H., Kim K.S. (2021). Recent Advances in Transdermal Drug Delivery Systems: A Review. Biomater. Res..

[3] Alkilani A.Z., Nasereddin J., Hamed R., Nimrawi S., Hussein G., Abo-Zour H., Donelly R.F. (2022). Beneath the Skin: A Review of Current Trends and Future Prospects of Transdermal Drug Delivery Systems. Pharmaceutics.

[4] Brunaugh A.D., Smyth H.D.C., Williams R.O. (2019). Topical and Transdermal Drug Delivery. Essential Pharmaceutics.

[5] Ramadon D., McCrudden M.T.C., Courtenay A.J., Donnelly R.F. (2021). Enhancement Strategies for Transdermal Drug Delivery Systems: Current Trends and Applications. Drug Deliv. Transl. Res..

[6] Potts R.O., Guy R.H. (1992). Predicting Skin Permeability. Pharm. Res..

[7] Prausnitz M.R., Langer R. (2008). Transdermal Drug Delivery. Nat. Biotechnol..

[8] Hadgraft J. (2001). Invited Review Skin, the Final Frontier. Int. J. Pharm..

[9] Pires P.C., Fernandes M., Nina F., Gama F., Gomes M.F., Rodrigues L.E., Meirinho S., Silvestre S., Alves G., Santos A.O. (2023). Innovative Aqueous Nanoemulsion Prepared by Phase Inversion Emulsification with Exceptional Homogeneity. Pharmaceutics.

[10] Shaker D.S., Ishak R.A.H., Ghoneim A., Elhuoni M.A. (2019). Nanoemulsion: A Review on Mechanisms for the Transdermal Delivery of Hydrophobic and Hydrophilic Drugs. Sci. Pharm..

[11] Marques L., Vale N. (2022). Salbutamol in the Management of Asthma: A Review. Int. J. Mol. Sci..

[12] Murthy S.N., Hiremath S.R. (2004). Clinical Pharmacokinetic and Pharmacodynamic Evaluation of Transdermal Drug Delivery Systems of Salbutamol Sulfate. Int. J. Pharm..

[13] Esen Yigit Ö., Lamprecht A. (2026). Impact of Drug Hydrophilicity on Transdermal Delivery by Nanoemulsions. Pharmaceutics.

[14] Todo H. (2017). Transdermal Permeation of Drugs in Various Animal Species. Pharmaceutics.

[15] Jacobi U., Kaiser M., Toll R., Mangelsdorf S., Audring H., Otberg N., Sterry W., Lademann J. (2007). Porcine Ear Skin: An in Vitro Model for Human Skin. Ski. Res. Technol..

[16] Lamprecht A., Bouligand Y., Benoit J.P. (2002). New Lipid Nanocapsules Exhibit Sustained Release Properties for Amiodarone. J. Control. Release.

[17] Cortés H., Hernández-Parra H., Bernal-Chávez S.A., Del Prado-Audelo M.L., Caballero-Florán I.H., Borbolla-Jiménez F.V., González-Torres M., Magaña J.J., Leyva-Gómez G. (2021). Non-Ionic Surfactants for Stabilization of Polymeric Nanoparticles for Biomedical Uses. Materials.

[18] Kaszuba M., Corbett J., Watson F.M., Jones A. (2010). High-Concentration Zeta Potential Measurements Using Light-Scattering Techniques. Philos. Trans. R. Soc. A Math. Phys. Eng. Sci..

[19] Tantra R., Schulze P., Quincey P. (2010). Effect of Nanoparticle Concentration on Zeta-Potential Measurement Results and Reproducibility. Particuology.

[20] Su R., Fan W., Yu Q., Dong X., Qi J., Zhu Q., Zhao W., Wu W., Chen Z., Li Y. (2017). Size-Dependent Penetration of Nanoemulsions into Epidermis and Hair Follicles: Implications for Transdermal Delivery and Immunization. Oncotarget.

[21] Mohamadi Saani S., Abdolalizadeh J., Zeinali Heris S. (2019). Ultrasonic/Sonochemical Synthesis and Evaluation of Nanostructured Oil in Water Emulsions for Topical Delivery of Protein Drugs. Ultrason. Sonochem..

[22] Chiu C.S., Huang P.H., Chan Y.J., Li P.H., Lu W.C. (2024). D-Limonene Nanoemulsion as Skin Permeation Enhancer for Curcumin Prepared by Ultrasonic Emulsification. J. Agric. Food Res..

[23] Souto E.B., Fangueiro J.F., Fernandes A.R., Cano A., Sanchez-Lopez E., Garcia M.L., Severino P., Paganelli M.O., Chaud M.V., Silva A.M. (2022). Physicochemical and Biopharmaceutical Aspects Influencing Skin Permeation and Role of SLN and NLC for Skin Drug Delivery. Heliyon.

[24] Binder L., Mazál J., Petz R., Klang V., Valenta C. (2019). The Role of Viscosity on Skin Penetration from Cellulose Ether-Based Hydrogels. Ski. Res. Technol..

[25] Bouwstra J.A., Honeywell-Nguyen P.L., Gooris G.S., Ponec M. (2003). Structure of the Skin Barrier and Its Modulation by Vesicular Formulations. Prog. Lipid Res..

[26] Rawlings A.V., Harding C.R. (2004). Moisturization and Skin Barrier Function. Dermatol. Ther..

[27] Elias P.M. (2005). Stratum Corneum Defensive Functions: An Integrated View. J. Investig. Dermatol..

[28] Hua S. (2015). Lipid-Based Nano-Delivery Systems for Skin Delivery of Drugs and Bioactives. Front. Pharmacol..

[29] BASF (2018). Technical Information:Kolliphor ^®^ EL.

[30] BASF (2019). Technical Information:Kolliphor ^®^ HS 15.

[31] Perrino C., Lee S., Spencer N.D. (2024). Quantitative Comparison of the Hydration Capacity of Surface-Bound Dextran and Polyethylene Glycol. Langmuir.

[32] Michaels A.S., Chandrasekaran S.K., Shaw J.E. (1975). Drug Permeation through Human Skin: Theory and Invitro Experimental Measurement. AIChE J..

[33] Miller J.M., Beig A., Krieg B.J., Carr R.A., Borchardt T.B., Amidon G.E., Amidon G.L., Dahan A. (2011). The Solubility-Permeability Interplay: Mechanistic Modeling and Predictive Application of the Impact of Micellar Solubilization on Intestinal Permeation. Mol. Pharm..

[34] de Oliveira Rangel Yagui C., Lineu Prestes A., Rangel-Yagui C.O., Pessoa A., Costa Tavares L. (2005). Micellar Solubilization of Drugs. J. Pharm. Pharm. Sci..

[35] Cuiné J.F., Mcevoy C.L., Charman W.N., Pouton C.W., Edwards G.A., Benameur H., Porter C.J.H. (2008). Evaluation of the Impact of Surfactant Digestion on the Bioavailability of Danazol after Oral Administration of Lipidic Self-Emulsifying Formulations to Dogs. J. Pharm. Sci..

[36] Silva C.L., Topgaard D., Kocherbitov V., Sousa J.J.S., Pais A.A.C.C., Sparr E. (2007). Stratum Corneum Hydration: Phase Transformations and Mobility in Stratum Corneum, Extracted Lipids and Isolated Corneocytes. Biochim. Biophys. Acta Biomembr..

[37] Biondo N.E., Argenta D.F., Caon T. (2023). A Comparative Analysis of Biological and Synthetic Skin Models for Drug Transport Studies. Pharm. Res..

[38] Lademann J., Richter H., Schaefer U.F., Blume-Peytavi U., Teichmann A., Otberg N., Sterry W. (2006). Hair Follicles—A Long-Term Reservoir for Drug Delivery. Skin. Pharmacol. Physiol..

[39] Otberg N., Patzelt A., Rasulev U., Hagemeister T., Linscheid M., Sinkgraven R., Sterry W., Lademann J. (2008). The Role of Hair Follicles in the Percutaneous Absorption of Caffeine. Br. J. Clin. Pharmacol..

