# Supplementary material for: Transdermal Delivery of Salbutamol from Nanoemulsions: Influence of Ternary Composition and Surfactant Architecture
Source: Pharmaceutics. 2026 Sep 8;18(9):1127. doi: 10.3390/pharmaceutics18091127 (PMC13610197; doi:10.3390/pharmaceutics18091127)
Supplement: Supplementary file 1 [file pharmaceutics-18-01127-s001.zip › pharmaceutics-4445569-supplementary.pdf]

Supplementary Materials

Transdermal Delivery of Salbutamol from Nanoemulsions: Influence of Ternary Composition and Surfactant Architecture

Özge Esen Yigit[1] and Alf Lamprecht[1,2,*]

[1] Department of Pharmaceutics, Institute of Pharmacy, University of Bonn, Gerhard-Domagk-Str. 3, 53121 Bonn, Germany

[2] Université Marie et Louis Pasteur, EFS, INSERM UMR1098 RIGHT, Besançon, F-25000, France

- Correspondence: alf.lamprecht@uni-bonn.de

Journal: Pharmaceutics
Manuscript ID: pharmaceutics-4445569

The supplementary materials include the following contents:

1. **Table S1.** Compositional design of the nanoemulsion formulations explored in this study.
2. **Table S2.** Descriptive summary of ATR-FTIR-derived lipid- and protein-associated parameters following nanoemulsion treatment of pig and mouse skin.
3. **Table S3.** Descriptive ATR-FTIR-derived lipid-associated parameters from exploratory human-skin experiments.
4. **Figure S1**. Representative FTIR CH-stretching spectra of human skin following treatment with nanoemulsion formulations containing PCO or PHS.
5. **Table S4.** Descriptive ATR-FTIR-derived lipid- and protein-associated spectral parameters from excipient-only reference experiments in pig and mouse skin.

Table S1. Compositional design of the nanoemulsion formulations explored in this study. Thirty-six formulations were prepared by systematically varying the relative proportions of water, PCO/PHS, and MCT oil (Miglyol® 812) across the ternary phase space. The percentages shown correspond to the vehicle composition excluding fixed components. All formulations contained 2.5% (w/w) API, 1% (w/w) lecithin, and 0.9% (w/w) NaCl, with a total batch size of 5 g.

Batch	Water (%)	PCO/ PHS (%)	MCT (%)
**1**	80	10	10
**2**	70	20	10
**3**	60	30	10
**4**	50	40	10
**5**	40	50	10
**6**	30	60	10
**7**	20	70	10
**8**	10	80	10
**9**	70	10	20
**10**	60	20	20
**11**	50	30	20
**12**	40	40	20
**13**	30	50	20
**14**	20	60	20
**15**	10	70	20
**16**	60	10	30
**17**	50	20	30
**18**	40	30	30
**19**	30	40	30
**20**	20	50	30
**21**	10	60	30
**22**	50	10	40
**23**	40	20	40
**24**	30	30	40
**25**	20	40	40
**26**	10	50	40
**27**	40	10	50
**28**	30	20	50
**29**	20	30	50
**30**	10	40	50
**31**	30	10	60
**32**	20	20	60

**33**	10	30	60
**34**	20	10	70
**35**	10	20	70
**36**	10	10	80

Table S2. Descriptive summary of ATR-FTIR-derived lipid- and protein-associated parameters following nanoemulsion treatment of pig and mouse skin. Average ν(CH_2 sym) stretching frequencies (2850 cm^{-1} region), CH_2/CH_3 ratios, and A(CH)/Amide II ratios are presented for PBS-exposed control skin and selected nanoemulsion formulations (9, 19, and 21) containing different water–surfactant–MCT compositions. Formulation 9 represents a water-rich system (70:10:20), formulation 19 an intermediate composition (30:40:30), and formulation 21 a surfactant-rich system (10:60:30). Values represent arithmetic means calculated from the available individual skin-section observations within each condition. The number of available sections ranged from 3 to 6 per condition; these values represent section-level observations rather than independent animals. Three spectra were recorded from each permeation-exposed skin section and averaged to obtain one section-level value.

Skin	Surfactant	Batch	CH_2 sym Stretching (2850 cm^{-1})	A(CH_2)	A(CH_3)	CH_2/CH_3 Ratio	A(Amide II)	A(CH)/A(Amide II) Ratio
**Mouse**	PCO	Control	2852.48	5.42	2.55	2.13	2.79	3.37
**Mouse**	PCO	9	2853.11	6.34	3.05	2.09	2.25	4.64
**Mouse**	PCO	19	2852.94	4.86	2.48	1.83	2.88	3.98
**Mouse**	PCO	21	2853.64	5.10	3.40	1.50	1.73	5.59
**Mouse**	PHS	Control	2852.34	5.48	3.05	1.90	2.80	3.51
**Mouse**	PHS	9	2852.50	6.31	3.33	1.93	2.02	5.27
**Mouse**	PHS	19	2853.32	3.52	2.45	1.64	2.13	4.38
**Mouse**	PHS	21	2853.11	4.38	2.09	2.10	1.14	6.33
**Pig**	PCO	Control	2852.45	0.85	0.58	1.45	4.79	0.29
**Pig**	PCO	9	2854.98	1.43	0.66	2.16	2.80	0.88
**Pig**	PCO	19	2854.61	1.39	0.74	1.90	2.83	0.79
**Pig**	PCO	21	2853.79	0.67	0.42	1.60	3.28	0.35
**Pig**	PHS	Control	2852.26	0.94	0.73	1.26	5.87	0.28
**Pig**	PHS	9	2853.33	2.54	1.39	1.71	4.11	1.15
**Pig**	PHS	19	2853.25	1.44	0.87	1.46	3.23	0.61
**Pig**	PHS	21	2853.39	1.17	0.86	1.37	3.98	0.46

Table S3. Descriptive ATR-FTIR-derived lipid-associated parameters from exploratory human-skin experiments. Human skin samples were additionally analyzed by ATR-FTIR after permeation experiments with selected nanoemulsion formulations containing PCO or PHS. Because salbutamol receptor-phase concentrations remained below the reliable quantification range under the present experimental conditions, the human-skin ATR-FTIR data were evaluated descriptively. The table summarizes selected lipid-related FTIR parameters of the human-skin sections.

Compared with mouse and pig skin, human skin exhibited only limited alterations in ν(CH_2) stretching vibrations and CH_2/CH_3 ratios following nanoemulsion treatment. The relatively small spectral shifts observed in the human-skin sections limited detectable changes in the evaluated CH-stretching parameters under the present experimental conditions. Values are presented as mean ± SD from three human skin sections per condition (n = 3 skin sections). Human-skin data were evaluated descriptively and were not subjected to inferential statistical analysis.

**Surfactant system**	**Formulation**	**ν (CH_2 sym) (cm^{-1})**	**ν (CH_2 asym) (cm^{-1})**	**A(CH_2)/A(CH_3)**
Control	PBS-exposed control	2851.54 ± 0.47	2921.51 ± 0.62	1.78 ± 0.23
PCO	F9	2851.96 ± 0.31	2922.12 ± 0.41	1.91 ± 0.17
PCO	F21	2852.11 ± 0.28	2922.37 ± 0.35	1.95 ± 0.14
PCO	F24	2852.06 ± 0.22	2922.21 ± 0.26	1.88 ± 0.16
PCO	F27	2852.18 ± 0.19	2922.42 ± 0.31	1.97 ± 0.12
PHS	F9	2852.09 ± 0.33	2922.12 ± 0.28	1.90 ± 0.13
PHS	F11	2852.17 ± 0.29	2922.06 ± 0.24	1.85 ± 0.12
PHS	F13	2852.24 ± 0.26	2922.18 ± 0.20	1.92 ± 0.11
PHS	F15	2852.31 ± 0.30	2922.30 ± 0.27	1.96 ± 0.15
PHS	F24	2852.42 ± 0.25	2922.41 ± 0.22	2.01 ± 0.14
PHS	F26	2852.51 ± 0.21	2922.53 ± 0.19	2.05 ± 0.12

Figure S1. Representative FTIR CH-stretching spectra of human skin following treatment with nanoemulsion formulations containing PCO or PHS. Normalized FTIR spectra of the CH-stretching region (2800–3000 cm^{-1}) obtained from human skin samples after permeation experiments with selected nanoemulsion formulations. Panel (A) shows formulations containing PCO, while panel (B) shows formulations containing PHS. Vertical dashed lines indicate the approximate positions of the ν(CH_2 sym) (~2850 cm^{-1}) and ν(CH_2 asym) (~2920 cm^{-1}) stretching vibrations. Spectra were normalized and vertically offset for clarity. Compared with the animal skin models, human skin exhibited only limited alterations in CH_2 stretching band positions and shapes following nanoemulsion treatment. Under the same experimental conditions, receptor-phase salbutamol concentrations remained below the LOQ. Three human skin sections were analyzed per condition (n = 3 skin sections); representative spectra are shown. Human-skin spectra were evaluated descriptively and were not subjected to inferential statistical analysis.

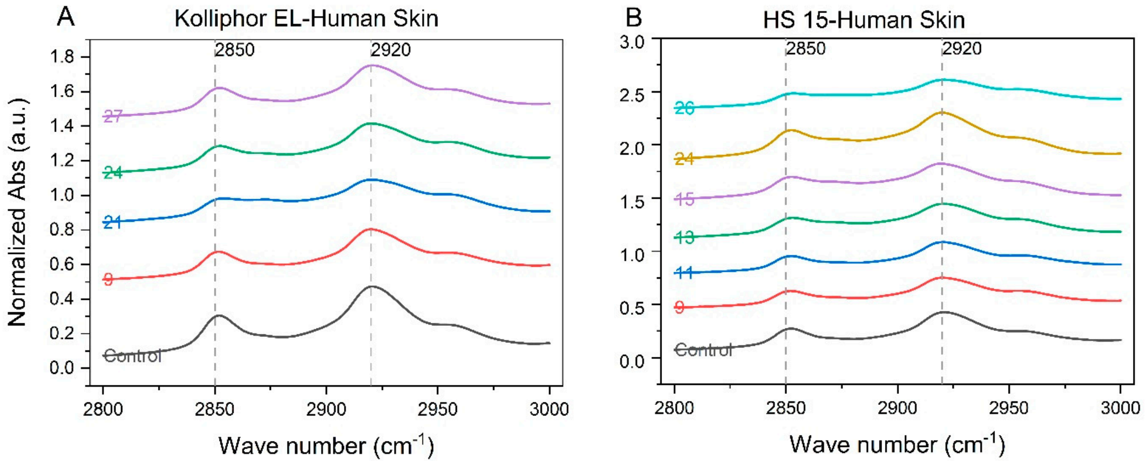

Table S4. Descriptive ATR-FTIR-derived lipid- and protein-associated spectral parameters from excipient-only reference experiments in pig and mouse skin. Pig and mouse skin samples were exposed separately to the aqueous phase (0.9% w/w NaCl), MCT (Miglyol® 812), PCO, or PHS under the same experimental conditions used for nanoemulsion-treated samples, washed with PBS, and dried prior to ATR-FTIR analysis. Reported values represent mean ν(CH_2) stretching frequencies (cm^{-1}) and absorbance areas integrated for A(CH_2), A(CH_3), A(CH_2)/A(CH_3), A(Amide II), and the relative A(CH)/A(Amide II) ratio. PBS-exposed control values are included to permit direct comparison with the corresponding excipient-treated samples. Values represent arithmetic means obtained from two individual skin samples per condition (n = 2). These experiments were exploratory and were evaluated descriptively.

Skin	Treatment	CH_2 sym Stretching (2850 cm^{-1})	A(CH_2)	A(CH_3)	A(CH_2)/A(CH_3)	A(Amide II)	A(CH)/A(Amide II)
Pig	PBS control	2851.57	0.69	0.21	3.31	2.97	0.31
Pig	Aqueous phase (0.9% NaCl)	2852.26	0.31	0.18	1.75	5.27	0.09
Pig	MCT	2852.72	0.28	0.16	1.76	4.96	0.09
Pig	PCO	2853.46	0.82	0.49	1.67	3.91	0.36
Pig	PHS	2853.13	0.98	0.50	1.98	4.05	0.36
Mouse	PBS control	2851.74	1.77	0.82	2.16	5.46	0.49
Mouse	Aqueous phase (0.9% NaCl)	2853.04	6.63	3.04	2.18	2.53	4.18
Mouse	MCT	2853.49	6.60	3.06	2.15	0.84	16.50
Mouse	PCO	2850.94	1.23	0.86	1.44	5.64	0.38
Mouse	PHS	2852.50	4.67	2.26	2.07	4.36	1.59

Note: The excipient-only measurements showed descriptive spectral patterns that differed between the two skin models. In mouse skin, the aqueous phase and MCT were associated with comparatively high A(CH)/A(Amide II) values relative to the PBS-exposed control, whereas smaller or different patterns were observed in pig skin. Given the limited number of measurements, these findings were interpreted descriptively. The patterns observed after exposure to the individual components differed from those obtained with the complete nanoemulsions (Table S2), suggesting that the complete formulation response cannot be explained solely by the individual component measurements.
